# Circulating SOD2 Is a Candidate Response Biomarker for Neoadjuvant Therapy in Breast Cancer

**DOI:** 10.3390/cancers14163858

**Published:** 2022-08-10

**Authors:** Mercè Juliachs, Mireia Pujals, Chiara Bellio, Nathalie Meo-Evoli, Juan M. Duran, Esther Zamora, Mireia Parés, Anna Suñol, Olga Méndez, Alex Sánchez-Pla, Francesc Canals, Cristina Saura, Josep Villanueva

**Affiliations:** 1 Vall d’Hebron Institut of Oncology (VHIO), 08035 Barcelona, Spain; 2 Department of Medical Oncology, Vall d’Hebron University Hospital, 08035 Barcelona, Spain; 3 Genetics Microbiology and Statistics Department, Universitat de Barcelona, 08028 Barcelona, Spain; 4 Statistics and Bioinformatics Unit, Vall d’Hebron Research Institute (VHIR), 08035 Barcelona, Spain; 5 Centro de Investigación Biomédica en Red de Cáncer (CIBERONC), 28029 Madrid, Spain

**Keywords:** manganese superoxide dismutase (SOD2), response biomarkers, breast cancer, secretome

## Abstract

**Simple Summary:**

The development of specific biomarkers to monitor response to the several available cancer drugs is a major challenge for modern clinical oncology. A reasonable solution for this problem could be to use circulating biomarkers linked to the tumor cell death induced by therapeutic treatment, instead of biomarkers linked to the molecular action of each drug. To test our hypothesis, we selected SOD2, an abundant mitochondrial protein extremely stable. During our studies, we proved that drug-induced tumor cell death increases the plasma levels of SOD2 correlating with the response to neoadjuvant therapy in breast cancer patients. We believe that measuring the circulating levels of SOD2 during therapeutic treatment of advanced cancer patients could offer a simple, non-invasive diagnostic tool complementing standard imaging techniques.

**Abstract:**

There is a great need for non-invasive tools that inform of an early molecular response to cancer therapeutic treatment. Here, we tested the hypothesis that proteolytically resistant proteins could be candidate circulating tumor biomarkers for cancer therapy. Proteins resistant to proteolysis are drastically under-sampled by current proteomic workflows. These proteins could be reliable sensors for the response to therapy since they are likely to stay longer in circulation. We selected manganese superoxide dismutase (SOD2), a mitochondrial redox enzyme, from a screening of proteolytic resistant proteins in breast cancer (BC). First, we confirmed the robustness of SOD2 and determined that its proteolytic resistance is mediated by its quaternary protein structure. We also proved that the release of SOD2 upon chemotherapy treatment correlates with cell death in BC cells. Then, after confirming that SOD2 is very stable in human serum, we sought to measure its circulating levels in a cohort of BC patients undergoing neoadjuvant therapy. The results showed that circulating levels of SOD2 increased when patients responded to the treatment according to the tumor shrinkage during neoadjuvant chemotherapy. Therefore, the measurement of SOD2 levels in plasma could improve the non-invasive monitoring of the therapeutic treatment in breast cancer patients. The identification of circulating biomarkers linked to the tumor cell death induced by treatment could be useful for monitoring the action of the large number of cancer drugs currently used in clinics. We envision that our approach could help uncover candidate tumor biomarkers to measure a tumor’s response to cancer therapy in real time by sampling the tumor throughout the course of treatment.

## 1. Introduction

The paucity of molecular diagnostic tools suitable for monitoring treatment outcomes is a major hurdle for the successful management of cancer patients [1]. There is a great need of tools that allow measuring the molecular response of tumors to cancer therapy in real time throughout the course of treatment [2]. Optimally, these tools should be non-invasive and they should reflect not only the size of the lesions but also the molecular response [3,4]. An early molecular response to treatment would allow clinicians to know whether a particular therapeutic choice is actually working. A treatment that does not work anymore not only does not help the patients, but it might also be contributing to the development of acquired resistance to the drug administered and probably to other cancer drugs. The earlier it is known that a treatment does not work, the faster the treatment can be stopped so the tumor cannot develop additional protective therapy escape mechanisms.

Despite most cancer diagnostic tools beyond imaging tests being based on measuring circulating proteins, the modern identification of useful circulating protein tumor biomarkers has been disappointing. In particular, mass spec proteomic efforts to identify useful tumor-derived circulating proteins using unbiased genome-wide approaches has demonstrated to be a daunting task plagued by limitations and controversy [5]. On one hand, tumor biology is extremely complex, cancer is a heterogenous group of diseases, and tumor-specific biomarkers are in low abundance in biological fluids. On the other hand, analytical techniques such as mass spectrometry cannot properly resolve the large dynamic range of protein concentrations in biological fluids, since tumor-derived proteins can only be enriched but not amplified. Alternative approaches including the profiling of secretomes, where tumor-derived proteins are enriched, with a follow-up validation in clinical samples have been attempted for tumor biomarker discovery [6,7].

Most mass-spec-based proteomic discovery workflows are based on the ability of trypsin to proteolyze proteins into peptides, i.e., bottom-up approach [8]. Subsequently, tryptic digests are analyzed by mass spec for different applications, including protein ID, quantification, and the characterization of post-translational modifications (PTM). Therefore, proteins that cannot be proteolyzed are basically undetectable by standard discovery-based proteomic approaches. However, there is no scientific reason why proteolysis-resistant proteins cannot be tumor biomarkers. In fact, the extreme stability of these proteins could contribute to their stability in plasma. Particularly, this group of resistant proteins could be amenable to the identification of biomarkers involving tumor cell death induced by cancer drugs.

In this work, we performed a screening of proteolytic resistant proteins in a breast cancer cell line secretome. The implementation of a modification of the standard proteomic workflow allowed us to profile a set of proteins that are extremely stable against proteolysis. We took one of them, manganese superoxide dismutase (MnSOD or SOD2), as a model protein for its validation as a candidate response biomarker for breast cancer neoadjuvant treatment. Neoadjuvant chemotherapy is becoming a preferred therapeutic option in breast cancer patients with locally advanced disease [9,10]. This clinical setting offers a homogenous group of patients and it is frequently used to assess new anti-cancer drugs and to evaluate candidate tumor biomarkers [11]. SOD2 is part of the superoxide dismutase family of redox enzymes that metabolize superoxide radicals into hydrogen peroxide, and it is located in the mitochondrial matrix of cells [12]. SOD2 is a protein overexpressed in cancer, probably because it alleviates the molecular damage induced by the increase in oxidative stress that tumor cells experience [13,14]. However, the expression of SOD2 seems to be stage-dependent. The expression of this protein is low in early-stage cancer while its expression increases in advanced cancer, particularly in metastasis [15]. Here, we carried out the functional validation of SOD2 as a response biomarker for chemotherapy, first in vitro, and then using plasma from BC patients undergoing neoadjuvant chemotherapy.

## 2. Materials and Methods

### 2.1. Cell Culture, Treatments, and Cell Titer

The breast cancer cell lines used in this project were purchased from ATCC and maintained at 37 °C in 5% CO_2_ and 95% humidified atmosphere air. Specifically, MDA-MB-231 and MCF7 cell lines were maintained in Dulbecco’s modified Eagle’s medium (DMEM/F12; Invitrogen, Waltham, MA, USA), and BT549 cells in RPMI medium (Invitrogen). All cell lines were supplemented with 10% fetal bovine serum (FBS, Invitrogen) and 2 mM L-glutamine (Invitrogen). When needed, cell lines were treated with paclitaxel (4 nM) or doxorubicin (30 nM or 2 µM). To generate a BT549 subline resistant to paclitaxel (BT549-DR cells), BT549 parental cells were treated chronically with 4 nM paclitaxel until they became resistant to the drug and started proliferating (Figure S2). The concentration to kill and/or inhibit growth of cells by 50% (IC_50_) of these drugs was quantified from drug–response curves by using CellTiter colorimetric assay analysis. The different cell lines were plated with four replicates for each condition (5000 cells/well) and after overnight incubation, drug treatment started. Specifically, the following drug concentrations were used: paclitaxel (1–8 nmol/L) and doxorubicin (1 nmol/L–2 μmol/L). Cell viability was measured after 72 h using the CellTiter assay (CellTiter-Blue Cell Viability Assay; Promega, Madison, WI, USA).

### 2.2. Molecular Cloning of SOD2 Mutants

His6-tagged human SOD2 cDNA sequence was cloned in the pcDNA 3.1 vector. The mutant Ile-58Thr-SOD2 was cloned in pcDNA3.1 and transfected in BT549-shSOD2 cells, specially designed for not silencing the mutant form. For the creation of shRNA-resistant versions of the cDNA, sequence 5′-CAGCCTGCACTGAAGTTCA-3′ was mutated to 5′-CAACCAGCTCTCAAATTTA-3′ to render an mRNA product insensitive to the shRNA expressed constitutively in the BT549 shSOD2 cells (target sequence CAGCCTGCACTGAAGTTCA). shRNA-resistant versions of SOD2 plasmids were transfected in BT549 cells expressing shSOD2 using FugeneHD (Roche, Basel, Switzerland) and following manufacturer recommendations. Briefly, 2.8 µg of DNA was added in 129 µL total volume of OptiMEM (Invitrogen). Next, 8.3 uL of FuGENE HD reagent was added and mixed by pipetting. The mix was incubated for 5–10 min at RT and 125 µL of complex was added to the cells seeded in a 100 mm plate containing 2 mL of fresh medium. After 48 h of transfection, medium was substituted with fresh growth medium containing 500 µg/mL of G418 (Gibco, Toronto, ON, Canada) to select for stably transfected clones. Cells were subsequently cultured for 12 days more, until non-transfected cells died, being subcultured at 1:5 ratio if needed.

### 2.3. Secretome Collection

Secretomes were prepared as previously described [16]. Briefly, 4 × 10^6^ cells in exponential phase were seeded in 150 mm tissue culture plates and allowed to grow for 48 h. After that, media was aspirated, and cells were washed five times: two times with PBS and the last three with serum-free media. After that, cells were maintained for the indicated time in the presence of serum-free media before collecting the conditioned media (secretome). The conditioned media were spun down at 200 g for 5 min, and the supernatants were collected and filtered through a Millex-GP 0.22 µM pore syringe driven filter (Millipore, Burlington, MA, USA). Then, secretomes were concentrated using a 10,000 MWCO Millipore Amicon Ultra (Millipore). Protein concentration was determined with a Pierce BCA protein assay kit (Thermo Scientific, Waltham, MA, USA).

### 2.4. In-Solution Digests

*Trypsin digestion in the presence of urea:* Secretome protein samples (15 µg of total protein), were taken to 40 µL of 6 M urea, and 50 mM ammonium bicarbonate by addition of the appropriate amount of lyophilized 8 M urea resulted in the 50 mM ammonium bicarbonate buffer. Samples were first reduced with DTT to a final concentration of 10 mM, for 1 h at RT, and then alkylated with 20 mM of iodoacetamide for 30 min at RT in the dark. Carbamidomethylation reaction was quenched by the addition of N-acetyl-L-cysteine to a final concentration of 35 mM followed by incubation for 15 min at RT in the dark. Samples were diluted with 50 mM ammonium bicarbonate to a final concentration of 1 M urea, modified porcine trypsin (Promega Gold) was added in a ratio of 1:10 (*w*/*w*), and the mixture was incubated overnight at 37 °C. The reaction was stopped with formic acid (FA) at a final concentration of 0.5% and the digest was kept at −20 °C until further analysis. 

*LysC/Trypsin digestion in the presence of guanidinium chloride*: Secretome protein samples (15 µg of total protein), pretreated or not as detailed above, were taken from 44 µL of 6 M guanidinium chloride, 50 mM ammonium bicarbonate. Samples were first reduced with DTT to a final concentration of 10 mM, for 1 h at 60 °C, and then alkylated with 20 mM of iodoacetamide for 30 min at RT in the dark. Carbamidomethylation reaction was quenched by addition of N-acetyl-L-cysteine to final concentration of 35 mM followed by incubation for 15 min at RT in the dark. Samples were diluted with 50 mM ammonium bicarbonate to a final concentration of 2 M guanidinium chloride, endoproteinase LysC from *Lysobacter enzymogenes* (Sigma, Kawasaki, Japan) was added in a ratio of 1:10 (*w*/*w*), and the mixture was incubated at 37 °C for 6 h. The digest was then diluted with 50 mM ammonium bicarbonate to a final concentration of 0.9 M guanidinium chloride, modified porcine trypsin (Promega Gold) was added in a ratio of 1:10 (*w*/*w*), and the mixture was incubated overnight at 37 °C. The reaction was stopped with formic acid (FA) at a final concentration of 0.5% and the digest was kept at −20 °C until further analysis.

### 2.5. Liquid Chromatography-Mass Spectrometry Analysis (LC-MS) and Protein Identification

Protein tryptic digests were analyzed using a linear ion trap Velos-Orbitrap mass spectrometer (Thermo Fisher Scientific, Bremen, Germany). Xcalibur software package was used to control the mass spectrometer, version 2.2.0 (Thermo Fisher Scientific, Bremen, Germany). Peptide mixtures were separated by on-line nanoflow liquid chromatography using an EASY-nLC 1000 system (Proxeon Biosystems, Thermo Fisher Scientific) with a two-linear-column system. Tryptic digests were first loaded on a trapping chromatographic column (Acclaim PepMap 100 nanoviper, 2 cm long, ID 75 μM and packed with C18, 3 μM particle size from Thermo Fisher Scientific) at 4 uL/min. Then, the peptide mixture was analyzed on the reversed-phase analytical column (Dr Maisch, 25 cm long, ID 75 μM, packed with Reprosil Pur C18-AQ, 3 μM particle size). Elution of peptides was carried out using 0.1% formic acid in water (mobile phase A) and acetonitrile with 0.1% formic acid (mobile phase B), with a linear gradient from 0 to 35% of mobile phase B for 120 min at a flow rate of 300 nL/min. Ions were produced by applying a voltage of 1.9kV to a stainless-steel nano-bore emitter (Proxeon, Thermo Fisher Scientific), attached to the end of the chromatographic column, on a Proxeon nano-spray flex ion source.

Data-dependent mode was used to operate the LTQ Orbitrap Velos mass spectrometer. A scan cycle started with a full-scan MS spectrum (from *m*/*z* 300 to 1600) obtained in the Orbitrap with setting a resolution of 30,000. The 20 most intense ions were targeted for collision-induced dissociation fragmentation in the linear ion trap when their intensity exceeded 1000 counts, dismissing singly charged ions. Collection of ions for both MS and MS/MS scans was achieved in the linear ion trap, and the AGC target values were set to 1 × 10^6^ ions for survey MS and 5000 ions for MS/MS scans. A maximum ion accumulation time was set to 500 and 200 ms in the MS and MS/MS modes, respectively. The normalized collision energy was set to 35%, and one microscan was obtained per each spectrum. Ions subjected to MS/MS with a relative mass window of 10 ppm were excluded from further sequencing for 20 s. A window of 20 ppm and isolation width of 2 Da was defined for all precursor masses. The lock mass option (*m*/*z* 445.120024) for survey scans was enabled in the Orbitrap measurements to improve mass accuracy.

### 2.6. Protein Identification and Quantitative Differential Analysis

Proteomic data were analyzed using the Proteome Discoverer v. 2.1 software (Thermo Fisher Scientific). Protein identification was performed using Mascot v. 2.5 (Matrix Science, London, UK) using the SwissProt database (2018_11, taxonomy limited to human proteins, 20,413 sequences). A precursor mass tolerance of 10 ppm was used to search the MS/MS spectra, fragment tolerance was set to 0.7Da, trypsin specificity was controlled by setting a maximum of 2 missed cleavages, cysteine carbamidomethylation was set as fixed peptide modification, and methionine oxidation as variable peptide modification. 

Protein identification files generated from Mascot (DAT files) were then loaded into the Scaffold software (version 3.00.07; Proteome software, Inc., Portland, OR, USA), resulting in a non-redundant list of identified proteins for each LC-MS/MS run per sample. Peptide identifications were validated whenever a PeptideProphet probability greater than 95% was achieved. Proteins identified with a probability higher than 95% and that contained at least two validated MS/MS spectra were accepted for further analysis. A false protein discovery rate (FDR) below 1.0%, as estimated by a database search, was achieved by using the described PeptideProphet and ProteinProphet tools. Using these filters, the files generated by the “Scaffold software” containing “spectral counts” (SpC) for each sample and their replicates were transferred to the R statistical environment. Exploratory data analysis was carried out by using principal component analysis (PCA) and hierarchical clustering of the SpC matrices to detect sample outliers and visualize patterns in the dataset. A GLM model based on the Poisson distribution was used to perform statistical modeling. During statistical analysis, an adjusted *p*-value < 0.05, a fold change >0.8 and minimum number spectral counts (SpC) >2 were set as thresholds [17,18].

### 2.7. Proteinase K Treatments

For Western blot analysis, 100 µg of secretome or 10 ng of recombinant protein (marca commercial) were digested with 20 µg/mL of Proteinase K (ThermoFisher) in a final volume of 20 µL. Samples were incubated at 37 °C with agitation O/N. Then, 1 µL of PMSF 100 nM was added to inactivate Proteinase K. For LC-MS/MS proteomic analysis, 100 µg of secretome was digested following the same protocol. Samples were filtered with 10 kDa amicon (Millipore) to remove the digested peptides since we were interested in analyzing the proteolytic-resistant proteins.

### 2.8. Western Blot

Cells were seeded in complete growth medium and allowed to grow at the specified times and conditions. Total protein extraction was performed using an NP-40-based lysis buffer. Protein quantitation and electrophoresis were performed as described elsewhere. Western blot analysis was performed with the following primary antibodies: mouse anti-alpha-tubulin (clone B-5-1-2, Sigma-Aldrich, St. Louis, MO, USA) used at 1:10,000, mouse anti-fibronectin (BD Cell Signalling, San Jose, CA, USA), SOD2 (sc-FL-222, Santa Cruz, Dallas, TX, USA) used at 1:1000, Histag (Cell Signaling), and cParp (Cell Signaling). Horseradish-peroxidase-conjugated secondary antibodies were sheep anti-mouse and donkey anti-rabbit IgG (GE Healthcare, Chicago, IL, USA) and used at 1:5000. All antibodies, unless otherwise stated, were used at 1:1000. Immunodetection was followed by visualization and densitometry using Image J software (Rasband, W.S., ImageJ 1.46r, U. S. National Institutes of Health, Bethesda, MD, USA). To evaluate the quaternary structure of SOD2, a native PAGE gel was performed, following the same protocol mentioned but without SDS.

### 2.9. SOD2 ELISA

SOD2 levels were measured by ELISA using the commercial kit (KA0528-Abnova) and following the manufacturer’s indicated protocol. 

### 2.10. SOD2 Stability in Human Active Serum

An amount of 7.5 µg of the BT549 secretome was mixed with human active serum in a final concentration of 4 µg/mL. The mix was split into 5 aliquots and incubated at 37 °C for 24 h, 48 h, 72 h, or 96 h. One sample of the mix without incubation was kept as the 0 h time point, as well as a sample of serum without secretome and the secretome as input. All samples were stored at −20 °C, and then SOD2 levels were analyzed by ELISA. 

### 2.11. SOD2 Immunoprecipitation

An amount of 100 µg of BT549 secretomes expressing SOD2-WT or SOD2-I58T with a HisTag were incubated with human active serum. The mix was split into 6 aliquots and incubated at 37 °C for 15′, 2 h, 7 h, 24 h, and 48 h. One sample of the mix was kept without any incubation and used as the 0 h control. Then, 20 µL of nickel beads/aliquot (Millipore), with a binding capacity of 1–5.5 μg of His-tagged protein per μL of bead suspension, were incubated with 500 µL of equilibration buffer (phosphate-buffered saline, 0.05% Tween-20, 10 mM imidazole, pH 8.0) for 1 min at RT with gentle mixing. The tubes were placed in the magnet rack, and after allowing the beads to migrate to the magnet the buffer was removed. The equilibrated beads were then added to each serum time point and incubated for 30 min at RT and afterwards 2 h at 4 °C, both with rotation. Then, samples were placed in the magnetic rack and serum was removed. The magnetic beads were then washed 10 times in 500 µL of Washing Buffer (PBS, 0.05% Tween-20, 20 mM imidazole, pH 8.0) with gentle mixing for 1′ at RT. Finally, proteins attached to the magnetic beads were eluted incubating the beads with Elution Buffer (PBS, 0.05% Tween-20, 300 mM imidazole, pH 8.0) for 15 min at RT with rotation. Tubes were then placed in the magnetic rack and the eluted fraction was collected, run though SDS-PAGE, and immunoblotted against SOD2. 

### 2.12. Patient Sample Collection

All plasma samples were obtained from Vall d’Hebron University Hospital (Barcelona, Spain). The study was approved by the hospital ethical committee, including a waiver of informed consent for the use of the samples for this project. An amount of 8 mL of venous blood was collected in an EDTA-BD Vacutainer (Beckton Dickinson, Franklin Lakes, NJ, USA). Blood was gently mixed by inverting 8 times to prevent clotting and immediately refrigerated in a vertical position. Tubes were then centrifuged at 1500 G for 15 min at 4 °C. Plasma was aliquoted in previously labeled and prechilled cryovials (Fisherbrand), and then stored at −80 °C. Repeated freeze–thaw cycles were avoided. Mammary tumors were measured using a caliper every three weeks. Tumor volume was calculated using the formula V = (W^2^ × L)/2 [19].

## 3. Results

### 3.1. Proteolysis-Resistant Secretome Screening as a Tumor Biomarker Discovery Approach

An optimal circulating protein biomarker should survive in the blood circulation system. Secreted proteins are designed to resist the harsh conditions outside cells, and hence they are good circulating candidate biomarkers, but their stability is likely to be heterogeneous. Since one of the most effective ways of removing proteins from circulation is proteolysis, we decided to perform a screening of proteolytic resistance in a cancer cell line secretome. First, we assayed the resistance to proteolysis of a secretome from BT549 cells, an invasive triple negative breast cancer (TNBC) cell line. We digested the secretome of BT549 with Proteinase K (Prot K), and the resulting secretome was analyzed by LC-MS/MS analysis together with a secretome control to identify the group of proteins that could resist the Proteinase K digestion. Next, a comparative proteomic analysis of the data obtained was conducted. Enrichment factors (Prot K/Control) were calculated for all the proteins of the analysis using normalized spectral counts. Unexpectedly, only nine proteins (ranging from 348% to 75% enrichment factors) survived the proteolysis with Prot K and were later identified by proteomic analysis (Figure S1).

**Figure 1:**
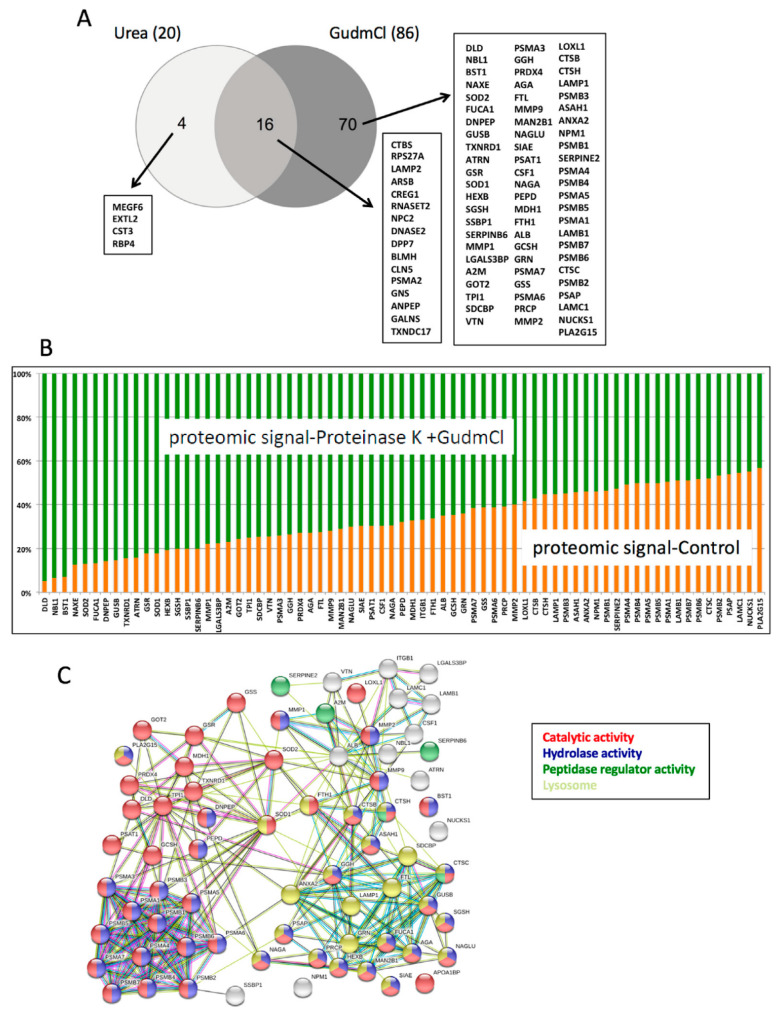
Proteolysis-resistant secretome screening as a tumor biomarker discovery approach. (**A**) Venn diagram showing an enrichment for a group of secretome proteins (labeled in the graph as GudmCl) that are not degraded by Prot K but need a harsh digestion protocol to be detected by LC-MS/MS. (**B**) Plot showing the different enrichment factors for the 70 proteins exclusively identified when using the Gu70-TrypLysC protocol. (**C**) STRING network analysis (https://string-db.org, accessed on 8 June 2020) of the 70 enriched proteins by Prot K digestion. The gene ontology analysis shows that most of these proteins are enzymes with hydrolase activity, or they reside in secretory granules and the lysosome.

We were surprised that only 9 secretome proteins out of 781 in the dataset were resistant to proteolysis with Prot K. Then, we reasoned that there could be another motive for finding such a small number of resistant proteins in our secretome. Given the extensive proteolytic treatment performed with Prot K in the secretome, the standard methodology to generate tryptic digests based on reduction, alkylation in the presence of a moderate concentration of urea might not be enough to digest the proteins that survived an extensive digestion with Prot K. To test this hypothesis, we devised a more denaturant protocol to generate protein digests out of cell line secretomes. This protocol, termed Gu70-TrypLysC, is based on the use of guanidinium chloride (GudmCl), high temperature (70 °C), and a double digestion (LysC followed by trypsin). We tested in parallel the two digestion protocols, the one based on urea and the one based on GudmCl, using equal amounts of a previously Prot K digested BT549 secretome (1:100 *w*/*w* protease/substrate). In this case, 20 proteins were identified after the Prot K digestion followed by the urea protocol, but 86 proteins were identified using the GudmCl protocol from a total dataset of 902 proteins (Figure 1A). These results suggest that there is a fraction of the secretome made of extremely stable proteins that are not detected by standard proteomic methodology. By performing the Prot K digestion and the GudmCl proteomic digestion, we enriched for a subset of very stable proteins (Figure 1B). The majority of these proteins are enzymes with hydrolase activity, and several of them reside either in secretory granules or in the lysosome (Figure 1C).

### 3.2. SOD2 as a Model for a Proteolytic-Resistant Protein

**Figure 2:**
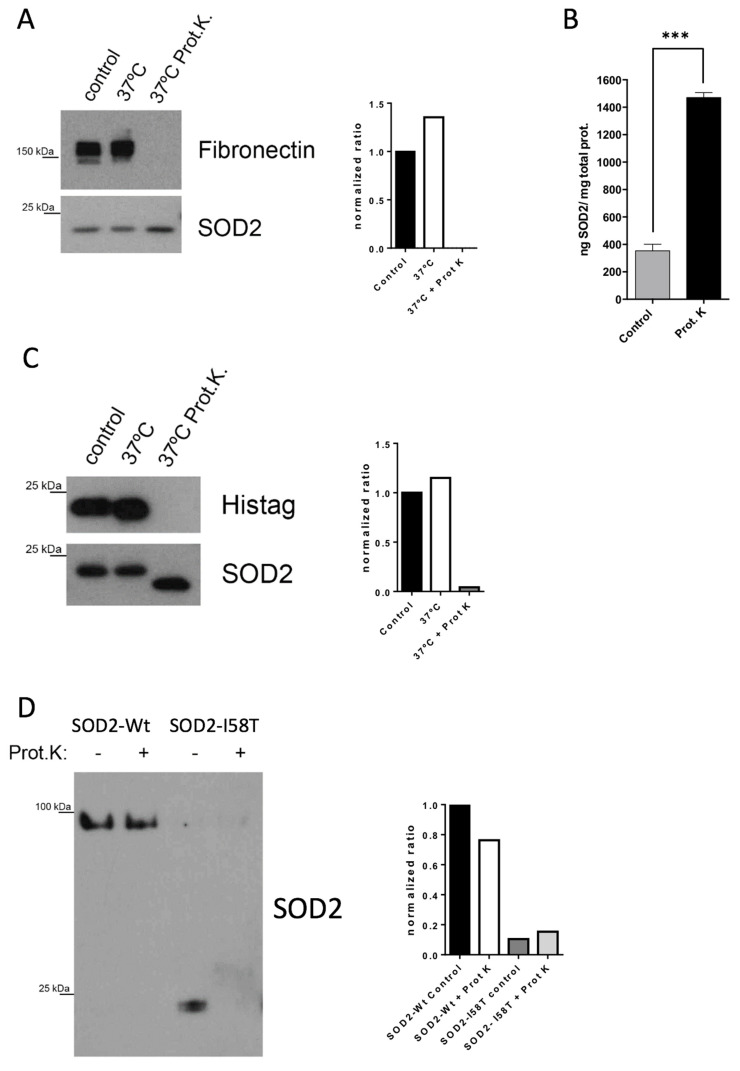
SOD2 is resistant to proteinase K degradation due to its quaternary structure. (**A**) The resistance of SOD2 to proteinase K activity was assessed by immunoblot in BT549 cells’ secretome and fibronectin was used as a degradation control. The secretomes were incubated at 37 °C o/n with or without Prot K, and a non-treated secretome was loaded as control. (**B**) SOD2 levels are also enriched in the digested BT549 secretome, when the samples are analyzed by ELISA. The mean of the triplicates and SD is shown. *** *p*-value < 0.001 (**C**) WB showing that SOD2 recombinant protein is resistant to Prot K activity, except for its His-tag that is digested after incubation with Prot K. (**D**) SOD2-I58T mutant is unable to form tetramers and is sensitive to Prot K. The secretomes from BT549 WT cells and BT549-shSOD2 -I58T cells were incubated with or without Prot K. Secretomes were loaded into native non-SDS-PAGE to evaluate the quaternary structure of the proteins. Quantification of the immunoblot bands was performed with Image J. Protein levels were normalized to the control condition of each experiment (Figure S4).

To prove our initial hypothesis, i.e., that proteins in the secretome that are proteolytically resistant and usually invisible to proteomic biomarker discovery efforts could make good tumor biomarkers, we selected a protein among the top resistant proteins. For the selection, we considered proteins with a large enrichment after Prot K digestion that are also overexpressed in breast cancer. Since our first proof of concept would be to test the candidate biomarker during the treatment with chemotherapy agents, we chose the BT549 cell line, which classifies in the TNBC subtype. TNBC is the most aggressive BC subtype, and it is usually treated with chemotherapy in the clinic. A protein that fulfilled our requirements is SOD2. Both SOD1 and SOD2 are largely enriched in our list, and are known to be robust proteins [20]. To confirm the proteomic results on SOD2, we repeated the Prot K digestion of a BT549 secretome, but this time using a WB readout. The results confirmed the resistance of SOD2 when equal amounts of secretome before and after Prot K digestion were loaded in SDS-PAGE (Figure 2A). While SOD2 was enriched, fibronectin—an abundant protein secreted by these cells—was proteolytically degraded. We obtained an additional confirmation of SOD2 enrichment in the secretome sample upon Prot K treatment using an ELISA readout (Figure 2B). We observed that the relative quantity of SOD2 to the total protein of the secretome sample was indeed higher when the secretome was digested with Prot K. To further confirm that the robustness of SOD2 is responsible for the enrichment observed in Figure 2A,B, we assayed a recombinant SOD2 with a His-tag in its C-terminus. Upon Prot K digestion the His-tag was removed, leaving the protein intact (Figure 2C). Next, we wanted to explore what confers the robustness of SOD2 against proteolysis. This protein is a homotetramer, where each monomer harbors a manganese ion coordinated to 3 His and 1 Asp amino acids of the protein [21,22]. To test whether the quaternary structure of SOD2 is related to its proteolytic resistance, we expressed a mutant of SOD2, Ile58Thr (I58T), in BT549 cells [23]. This mutation is a polymorphic variant of SOD2, where the tetramer structure is destabilized [23]. To avoid having the expression of both the WT and the I58T SOD2 forms at the same time, we expressed the mutant in BT549-shSOD2 cells designed to not silence the mutant form (see Section 2). To test our hypothesis, we studied the secretomes of BT549 cells expressing either the WT SOD2 protein or the I58T mutant using native gels and WB. The secretomes were also treated with Prot K to test their proteolytic resistance. The results show that while the WT protein runs at MW compatible with being a tetramer and it is resistant to proteolysis, the I58T mutant is a monomer that is sensitive to proteolysis with Prot K (Figure 2D).

### 3.3. The Release of SOD2 upon Chemotherapy Treatment Correlates with Cell Death in TNBC Cells

**Figure 3:**
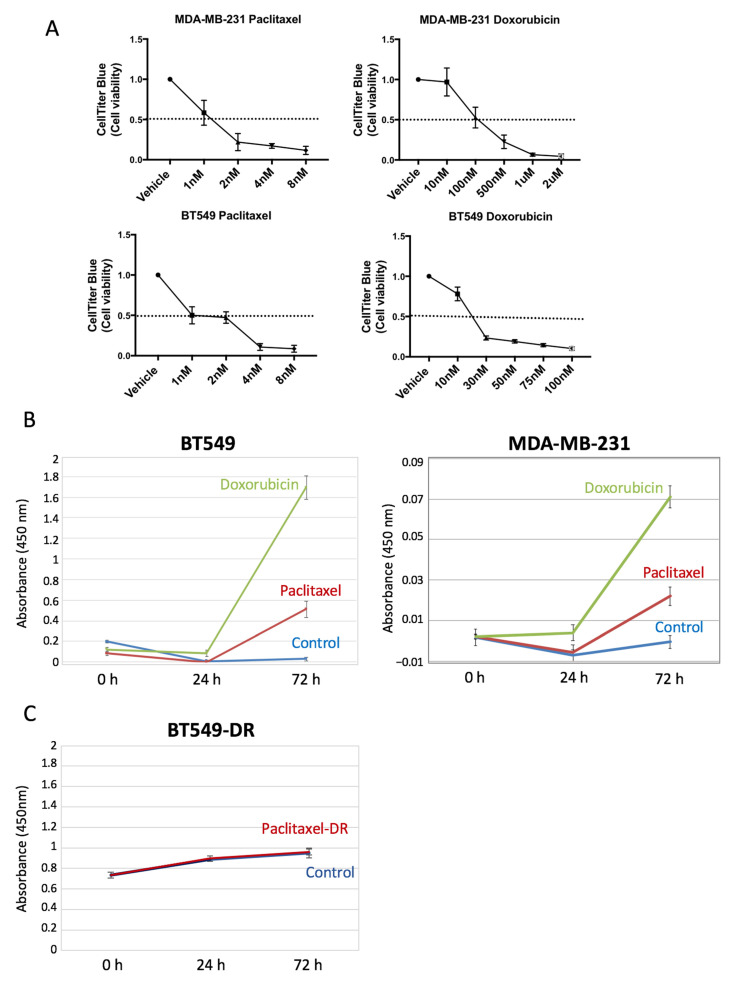
SOD2 is released when BrCa cells are treated with chemotherapy. (**A**) MDA-MB-231 and BT549 cells were treated with paclitaxel and doxorubicin for 3 days to induce cell death and CellTiter was performed to assess cell viability. (**B**) SOD2 levels in conditioned media from cells treated with paclitaxel 4 nM and doxorubicin (30 nM for BT549 left panel and 2 µM for MDA-MB-231 right panel) for 3 days were evaluated in triplicate measurements by ELISA. (**C**) SOD2 levels in conditioned media from BT549-DR cells treated with paclitaxel 4 nM were measured in triplicate by ELISA. Error bars correspond to the standard deviation from the mean of the triplicates.

SOD2 is a very abundant protein in the mitochondrial matrix of cells. We reasoned that being so robust to proteolysis and very abundant intracellularly, tumor cell death would cause a large increase in the levels of SOD2 in the supernatants of tumor cells treated with cytotoxic therapy. First, we set up assays with cytotoxic chemotherapeutic agents used in the clinic to treat BC patients in breast cancer cell lines. We used paclitaxel, a microtubule-targeting agent, and doxorubicin, an inhibitor of the topoisomerase 2, both widely used in the clinic to treat TNBC patients. To test whether SOD2 could be used as a surrogate circulating biomarker of cell death, we treated two TNBC cell lines (BT549 and MDA-MB-231) with the two chemotherapy agents and monitored cell death (Figure 3A). Then, using the same experimental setup, we took aliquots of the conditioned media at different time points during the cytotoxic treatment and measured SOD2 levels by ELISA. The results show an increase in the levels of SOD2 to the conditioned medium of tumor cells sensitive to chemotherapy treatment (Figure 3B). To further support our hypothesis that chemotherapeutic-sensitive BrCa cells release SOD2, and it can be used as a treatment response biomarker, we generated a resistant subline to paclitaxel from BT549 cells (BT549-DR cells) and measured the SOD2 levels in conditioned media (see Section 2). SOD2 levels measured upon cytotoxic therapy in BT549-DR cells were unchanged compared to vehicle-treated cells (Figure 3C). Therefore, this result confirms that the increased levels of SOD2 in parental cells treated with chemotherapy come from the death of tumor cells (Figure 3B). 

### 3.4. SOD2 Is Extremely Stable in Human Serum

**Figure 4:**
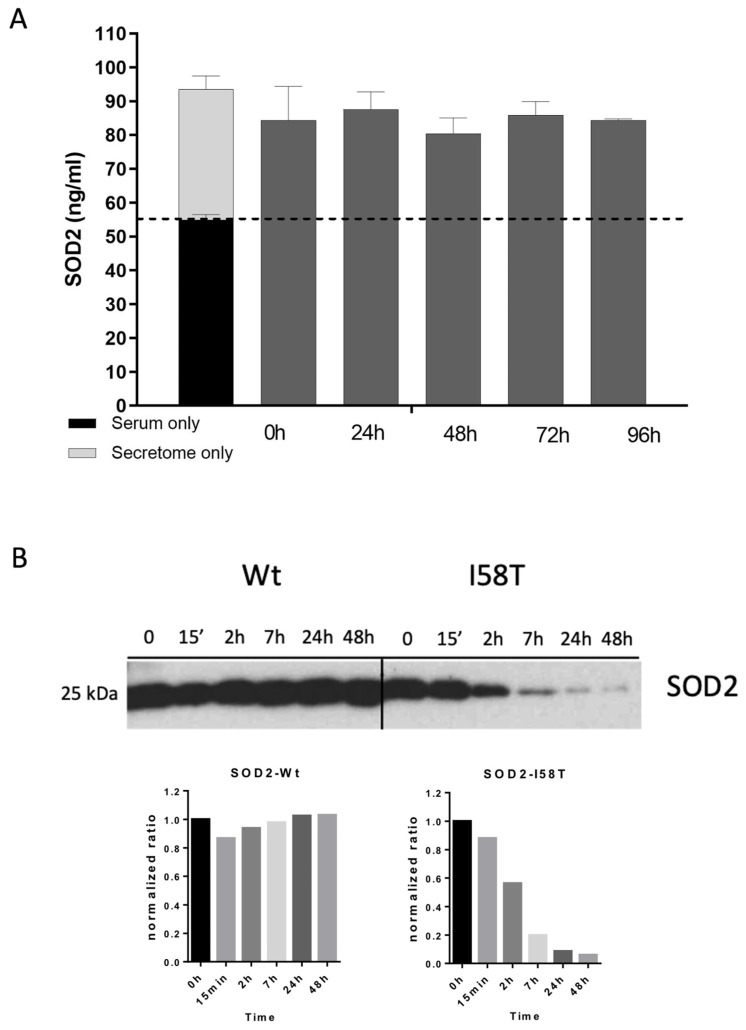
SOD2 is stable within human serum. (**A**) SOD2 levels were stable when incubated in human serum. Secretome from BT549 cells was spiked into human serum and incubated at 37 °C for 0 h (as initial control), 24 h, 48 h, 72 h, and 96 h. The levels of SOD2 were assessed by ELISA. The dashed line in the plot indicates the endogenous levels of SOD2 in the human serum used. (**B**) SOD2-I58T was degraded in human serum while SOD2-WT was resistant. Secretomes expressing WT-SOD2-histag or SOD2-I58T-histag were spiked into human serum and incubated up to 48 h. The two tagged proteins were purified using nickel beads at different time points (0 h, as control, 15′, 2 h, 7 h, 24 h, 48 h). An immunoblot was performed to detect SOD2 levels. Quantification of the immunoblot bands was performed with Image J. Protein levels were normalized to the control condition of each experiment.

Based on the in vitro results obtained up to this point, we decided to use circulating SOD2 as a candidate biomarker of cancer chemotherapy response. In preparation for the work with clinical samples, we first tested the stability of SOD2 in serum. We performed a kinetic study by spiking secretome from BT549 cells in human serum. The secretome–serum mix was incubated at 37 °C for 96 h, and SOD2 was measured by ELISA at different time points by taking equal aliquots from the mix (Figure 4A). The results showed no loss of SOD2 over the 4-day experiment. In order to have a positive control in our experiment, we decided to use the I58T- SOD2 mutant since it is sensitive to Prot K proteolysis (Figure 2D). Unfortunately, the SOD2-ELISA kit we use does not recognize the I58T mutant (Figure S3). Although we do not know for a fact, the mutation site could be part of the epitope recognized by the antibodies used in the kit. Alternatively, the ELISA kit might be recognizing the SOD2 tetramer, which the I58T mutant is not able to form (Figure 2D). To overcome the limitation of the ELISA kit, we took advantage of the His-tag present in the C-terminus of both the WT and the I58T proteins. Secretomes of BT549 cells expressing SOD2-WT and I58T were spiked into human serum, as we performed before with the secretome of BT549 cells. Then, at different time points of the experiment, equal fractions of the two spiked sera (WT and I58T) were enriched using magnetic nickel beads and immunoblotted for SOD2 (Figure 4B). The results clearly showed that while the WT-SOD2 protein is stable in serum for a long time, the I58T mutant is not, being degraded in a few hours. Collectively, these results suggest that SOD2 could be a good circulating biomarker.

### 3.5. Circulating SOD2 Is a Candidate Biomarker of Response to Tumor Cytotoxic Agents

**Figure 5:**
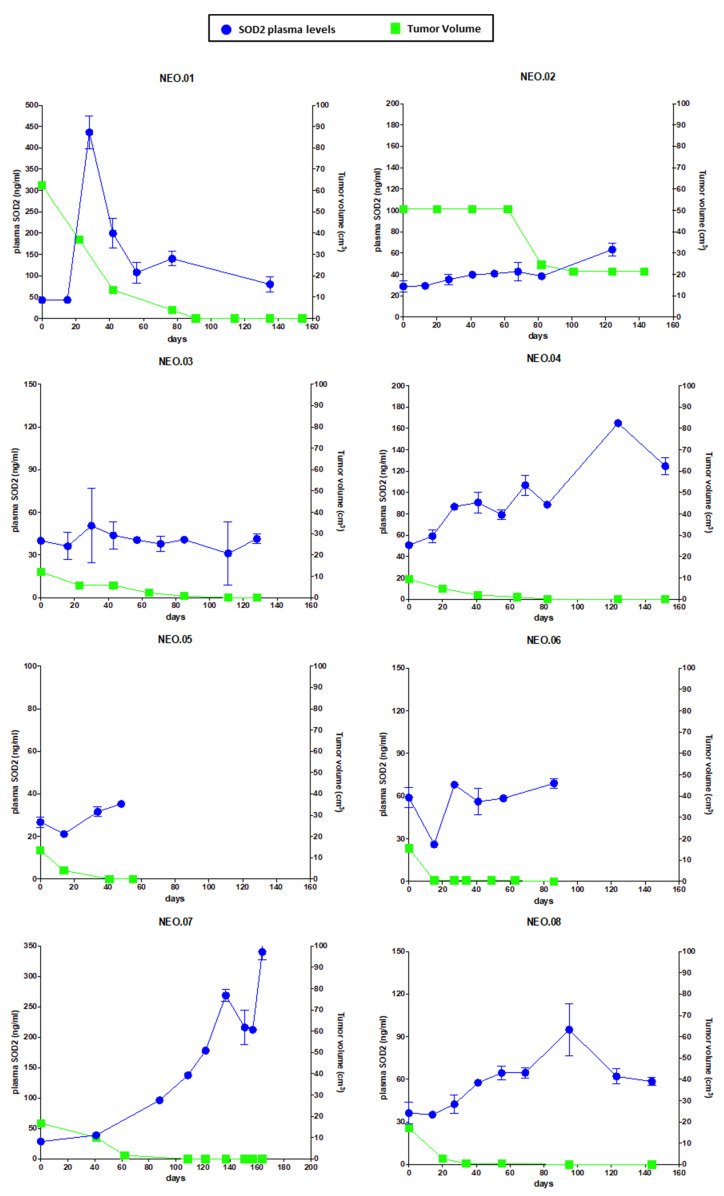
SOD2 levels in the plasma of BrCa patients correlate with their response to neoadjuvant chemotherapy. Plot showing SOD2 plasma levels and tumor volume over the time of neoadjuvant treatment. Tumor volumes (cm^3^) were assessed clinically by caliper measurement, and SOD2 levels were evaluated in triplicate by ELISA in plasma from 8 BrCa patients. Error bars correspond to the standard deviation from the mean of the triplicate measurements.

Next, we performed a clinical validation of SOD2 levels in patients diagnosed with early BC. We selected patients with triple-negative (TN) disease, defined as lack of expression of estrogen receptor, progesterone receptor, and human epidermal growth factor receptor 2 (HER2), and Luminal B HER2-negative intrinsic subtype undergoing neoadjuvant chemotherapy treatment based on taxanes and anthracyclines [24]. Some patients with TN disease had also received carboplatin concomitant to taxane treatment. SOD2 levels were measured by ELISA in plasma from BC patients (Table S1). Plasma samples were taken from patients before treatment and every 1 or 3 weeks depending on the drug regimen, usually for 6 months. Here, we present data for eight patients with a baseline sample (BL) and at least three samples collected during the treatment. All the treatment samples are correlated with the tumor response during neoadjuvant treatment using the size of tumor lesion at every clinical evaluation and calculating the tumor volume (Figure 5).

The results show that the samples obtained during the neoadjuvant treatment increased the circulating levels of SOD2 compared to BL samples when tumors were responding to the treatment. Therefore, this dataset suggests that the relationship between SOD2 plasma levels and tumor volume follows a negative correlation (Figure 5). Moreover, we observed different behaviors for the levels of SOD2 during the neoadjuvant treatment. One patient who achieved a quick clinical response presented an earlier elevation of SOD2 levels, independent of a chemotherapy agent and/or BC subtype (NEO.01). Similar results were observed for patients NEO.04 and NEO.08, where the levels of SOD2 increased correlating with the response to the drug, although slower than for NEO.01. Conversely, patients who presented a slow and progressive clinical response (NEO.02, NEO.03) had a less steep ascent in the curve of SOD2 levels. The treatment for patients NEO.05 and NEO.06 was stopped early because of toxicity. However, the trend of the curve followed by SOD2 levels at the time of finalizing the treatment was clearly ascending, and therefore following a negative correlation with respected of tumor size. Taking into account these results, we also included a patient diagnosed with Luminal A BC who underwent neoadjuvant treatment due to a locally advanced disease (cT3N0M0) and was a premenopausal woman (NEO.07). In this case, we also could also observe a negative correlation between tumor volume and SOD2 plasma levels. Therefore, our results suggest that circulating SOD2 is a candidate biomarker of drug response, although further work is needed to assess the full potential of this biomarker. We envision that the robustness of SOD2 could make of this protein an ideal circulating tumor biomarker for cytotoxic cancer therapies.

## 4. Discussion

Here, we tested the hypothesis that proteolytically resistant proteins could be candidate tumor biomarkers. Proteins resistant to proteolysis are likely to be relevant for tumor biomarker discovery and are drastically under-sampled by current proteomic workflows. The rationale of our approach is that upon tumor cell death, robust proteins will stay longer in circulation and provide a reliable sensor for the response to therapy. Most tumor-derived proteins are likely to be removed quickly from circulation, since they will not endure the physicochemical environment either by losing folding stability or being proteolyzed. Identifying highly stable tumor-derived proteins that enter into circulation could be a valuable approach to, for example, predict responses to cancer therapy. Our approach could complement other strategies relying on the tumor cell death induced by cancer drugs that are being tested to monitor the response to cancer drug therapy [25,26,27].

Several of the proteins enriched by our proteolytic assay are unconventionally secreted proteins, since their sequences do not have signal peptides directing them to the ER–Golgi secretory pathway. Unconventional secretion of theoretically intracellular proteins has been recently proven far more common than previously expected. Different unconventional secretion pathways relying on either extracellular vesicles or soluble proteins have been described [16,28,29]. Here, we chose to functionally validate SOD2 as a tumor biomarker. While thought to be a strict mitochondrial protein, SOD2 has been recently reported to be secreted in renal carcinoma [30]. Likewise, the other intracellular superoxide dismutase, SOD1, has also been described to be non-classically secreted in yeast [31]. Here, we suggest that SOD2 is also non-classically secreted in breast cancer cells. However, our current data suggest that the large increase in SOD2 levels measured by ELISA both in vitro (in tumor cells) and in vivo (on BC patients) would originate mostly from the mitochondrial form liberated upon cell death rather than from secretion.

Proteolytic activities have been previously proposed to generate new tumor biomarkers, for example in the form of patterns of the serum peptidome amplified by the proteolytic activities of tumor-derived proteases or by the proteolytic degradation of cytokeratin-18 by caspases during apoptosis [27,32]. In our case, we propose an in vitro approach to predict which tumor-derived proteins could be proteolytically resistant and hence stable in circulation during cancer therapy. The highly resistant proteins identified in this work are circumscribed to very specific cellular biochemistries. Most of these proteins are enzymes, and among them the majority are proteases, including proteasomal proteins, lysosomal proteases, cathepsins, and matrix metalloproteases. The second group of enzymes enriched by our proteolytic assay is made of redox enzymes functioning in different cellular organelles. A provocative idea derived from this work is that several of the stable proteins identified could potentially be candidate circulating cell death biomarkers. We believe that the dataset provided with this work contains other potential tumor biomarkers. However, parameters including protein solubility in plasma and clearance kinetics from circulation will play a role in the biomarker potential of each protein assayed.

We have demonstrated that the extreme proteolytic stability shown by SOD2 in tumor cells is due to its quaternary structure. The disruption of the tetramer structure greatly diminishes the stability of the protein both in cell culture and in plasma. The initial in vitro validation studies prove that extracellular levels of SOD2 in cell lines correlate with the tumor cell death induced by chemotherapy. Our results show that regardless of whether a tumor cell is sensitive or resistant to chemotherapy, the changes in the slope of the curve describing the levels of SOD2 correlate well with tumor cell death. In fact, the biomarker is the increase in the levels of SOD2, independent of the baseline levels. This hypothesis is demonstrated using BT549 and MDA-MB-231 cells (Figure 3B), which despite having different baseline levels of SOD2, show a significant increase in the SOD2 levels when treated with chemotherapeutic drugs. Furthermore, tumor cells resistant to paclitaxel do not show a change in the curve slope compared to their sensitive counterparts when treated, despite having higher baseline levels.

Next, we moved to perform a preliminary clinical validation of SOD2 as a response biomarker for chemotherapy treatment in BC patients. A remarkable finding in this work is that there is a correlation relationship between SOD2 plasma levels and tumor volume in a group of BC patients (TNBC and Luminal B HER2-negative) during neoadjuvant treatment. As shown in Figure 5, the reduction in tumor size in patients undergoing neoadjuvant therapy correlates with the changes in the circulating levels of SOD2, despite their different SOD2 baseline levels. Therefore, the measurement of SOD2 levels could improve the non-invasive monitoring of the therapeutic treatment in BC patients. Furthermore, beyond our proof-of-concept study, we believe that SOD2 levels should be explored in the treatment of patients with advanced cancer. A drug able to kill tumor cells in the metastatic setting would most likely cause an increase in the levels of circulating SOD2 as long as there is tumor cell death. Therefore, a circulating biomarker that correlates with response to therapy in advanced disease could improve the response evaluation using a quick and non-invasive assessment collected at the time of treatment administration, complementing standard imaging techniques such as computed tomography (CT) scans. Moreover, SOD2 levels could provide a cost-effective biomarker of response to the treatment in the first 2–3 months as a cell death BM, because these levels could identify whether the tumor is responding to this therapy, information which is not available through imaging techniques in this early phase of the treatment.

## 5. Conclusions

The large amount of cancer drugs and the different genomic alterations arising in tumors makes it almost impossible to develop specific biomarkers for each treatment. A plausible solution for these problems could be to develop circulating biomarkers linked to the tumor cell death induced by the treatment, instead of biomarkers linked to the molecular action of each drug. The results presented here show that tumor-derived proteolytically resistant proteins could be general drug response biomarkers that could monitor the treatment outcome. The measurements of circulating SOD2 in plasma could improve the non-invasive monitoring of the therapeutic treatment in breast cancer patients. We envision that our approach could help uncover candidate tumor biomarkers to measure a tumor’s response to cancer therapy in real time by sampling the tumor throughout the course of treatment.

## Data Availability

The data presented in this study are available on request from the corresponding author. Breast cancer cell lines MDA-MB-231, MCF7 and BT549 were purchased from the American Type Culture Collection (ATCC).

## Supplementary Materials

The following supporting information can be downloaded at: https://www.mdpi.com/article/10.3390/cancers14163858/s1, Table S1: Patients’ clinical data. Figure S1. Plot showing spectral count measurements comparing results from a secretome processed using the standard proteomic workflow with a protocol using a pretreatment with Proteinase K, Figure S2. Cell proliferation of BT549 cells resistant to paclitaxel, Figure S3. SOD2-I58T mutant is not recognized by ELISA. SOD2 levels were measured in two secretomes, from BT549-WT and BT549-shSOD2-I58T cells, by immunoblot (left panel) and by ELISA (right panel). Figure S4. Uncropped blots.
