# Supplementary material for: Circulating SOD2 Is a Candidate Response Biomarker for Neoadjuvant Therapy in Breast Cancer
Source: Cancers (Basel). 2022 Aug 10;14(16):3858. doi: 10.3390/cancers14163858 (PMC9405919; doi:10.3390/cancers14163858)

Supplementary Materials

Table S1. Patients' clinical data.

Figure S1. Plot showing spectral count measurements comparing results from a secretome processed using the standard proteomic workflow with a protocol using a pretreatment with Proteinase K.

Figure S2. Cell proliferation of BT549 cells resistant to paclitaxel.

Figure S3. SOD2-I58T mutant is not recognized by ELISA. SOD2 levels were measured in two secretomes, from BT549-WT and BT549-shSOD2-I58T cells, by immunoblot (left panel) and by ELISA (right panel).

Figure S4. Uncropped blots

Table S1

ID	Breast Cancer Subtype	Histologic grade	Ki67 (%)	cTNM	Neoadjuvant chemotherapy	Clinical response	Radiologic response [a]	Data IQ	ypTNM	Relapse
**Neo_1**	Luminal B HER2 -	2	30	T3N3M0	Paclitaxel > Antracyclines	CR	PR	4/11/2016	ypT1aN1mic	Yes
**Neo_2**	Luminal B HER2 - --> TN	2	45	T2N0M0	Paclitaxel > Antracyclines	PR	SD	4/21/2016	ypT2N0	No
**Neo_3**	Luminal B HER2 -	3	70	T2N0M0	Paclitaxel > Antracyclines	CR	CR	7/31/2018	ypT1bN0	No
**Neo_4**	TNBC	2	70	T2N1M0	Carboplatin-Paclitaxel > Antracyclines	CR	CR	9/7/2018	ypT0N0- pCR	No
**Neo_5**	TNBC	3	80	T2N0M0	Carboplatin-Nab-Paclitaxel (only 8C)	CR	CR	10/10/2018	ypT0N0- pCR	No
**Neo_6**	TNBC	3	95	T2N3M0	Carboplatin-Paclitaxel -> Myocet-Ciclo	CR	PR	8/28/2018	ypT0N0- pCR	Yes
**Neo_7**	Luminal A	2	15	T3N0M0	Antracyclines > Paclitaxel	CR	PR	11/23/2018	ypT1bN1 mic	No
**Neo_8**	TNBC	3	55	T2N0M0	Carboplatin-Paclitaxel > Antracyclines	CR	CR	11/7/2018	ypTisN0	No

Notes:

a: Radiologic response assessment to neoadjuvant chemotherapy was done to classify diasease outcome as a complete response (CR), partial response (PR), stable disease (SD) or progressive disease (PD) using the RECIST criteria v1.1.

Figure S1

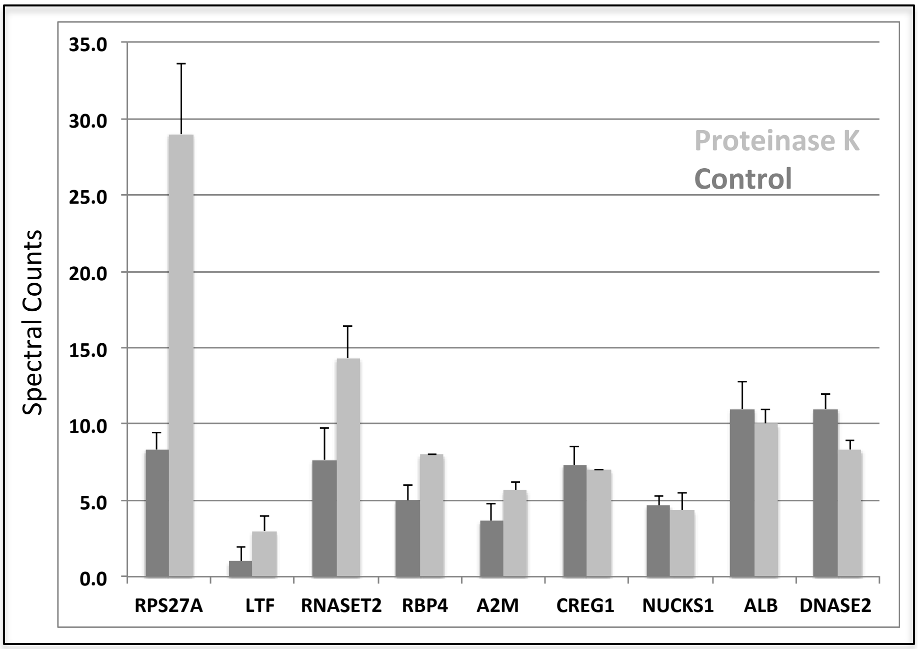
35.0
30.0
25.0
20.0
15.0
10.0
5.0
0.0
Spectral Counts
Proteinase K
Control
RPS27A
LTF
RNASET2
RBP4
A2M
CREG1
NUCKS1
ALB
DNASE2

Figure S2

BT549 cells resistant to Paclitaxel

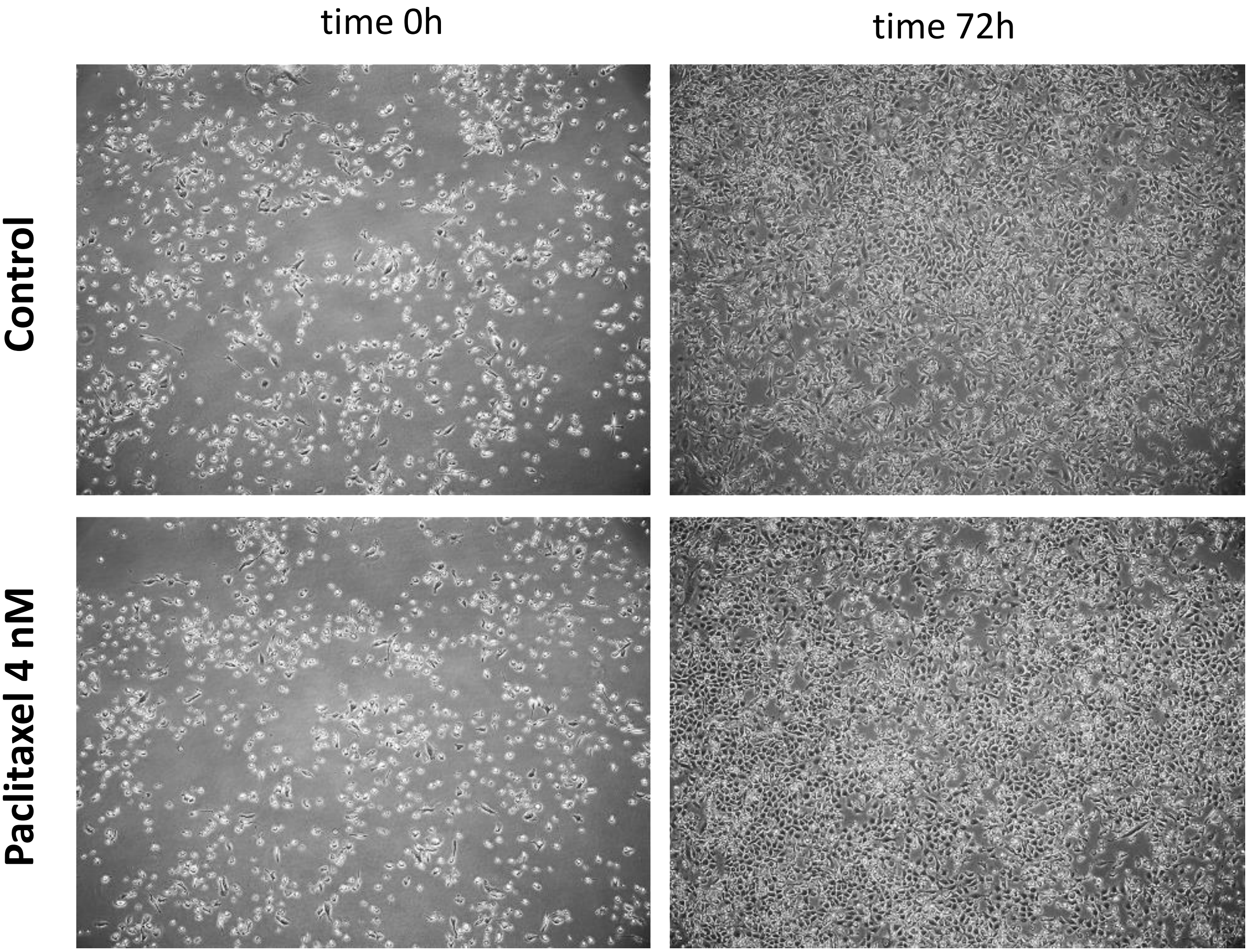

Figure S3

BT549 Secretomes

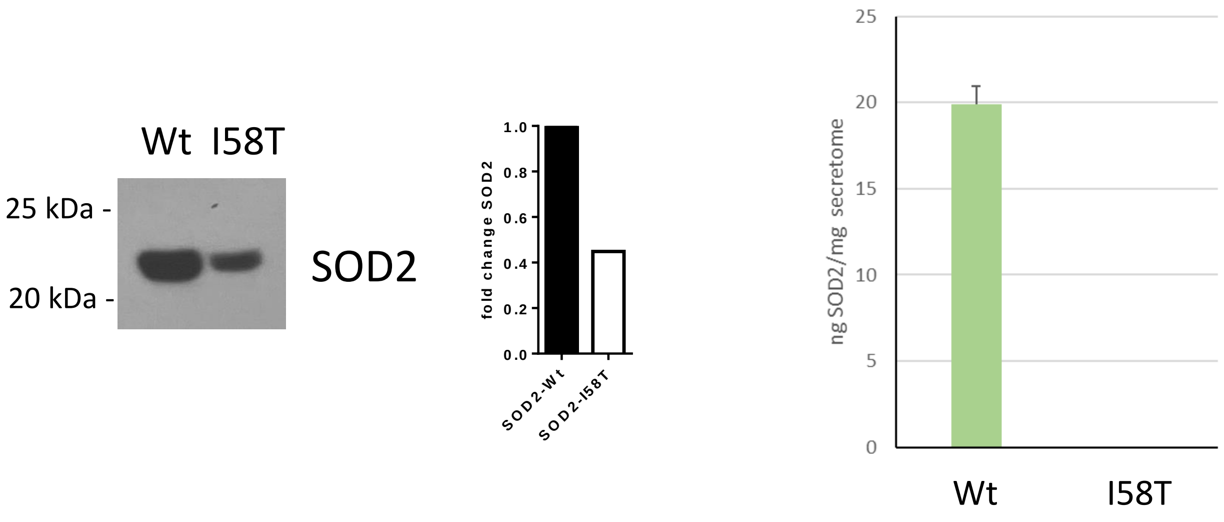

Figure S4

Uncropped blots

Figure 2A

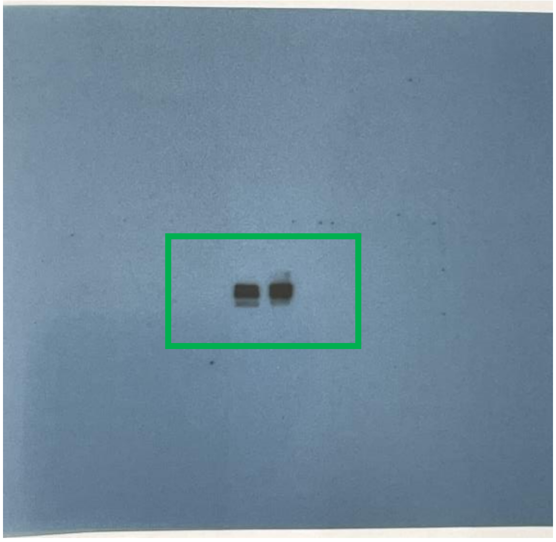

Figure 2A and 2C

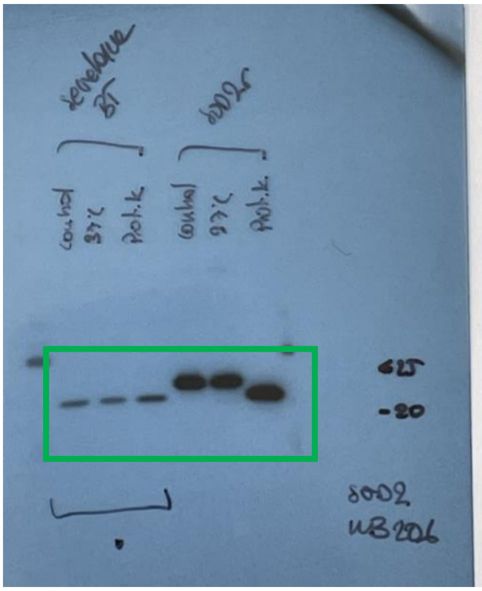

Figure 2C

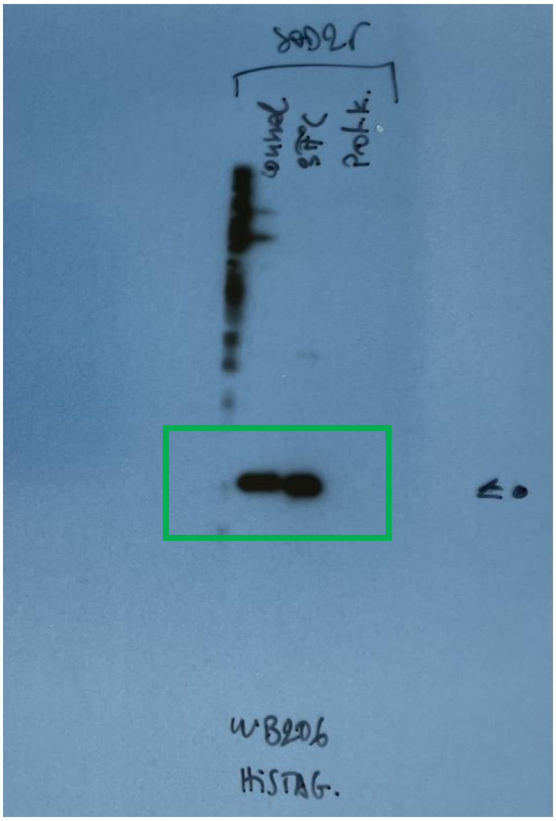

Figure 2D

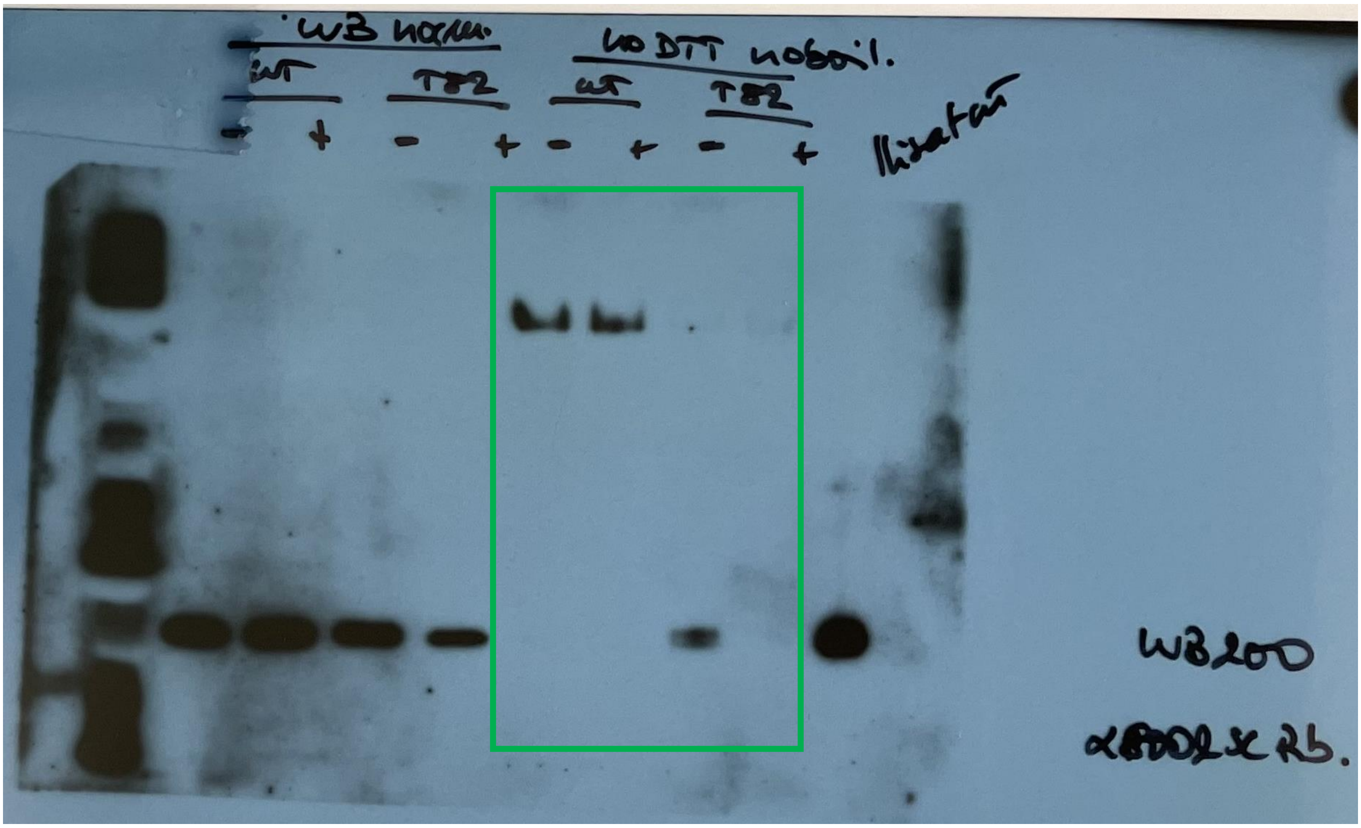

Figure S3

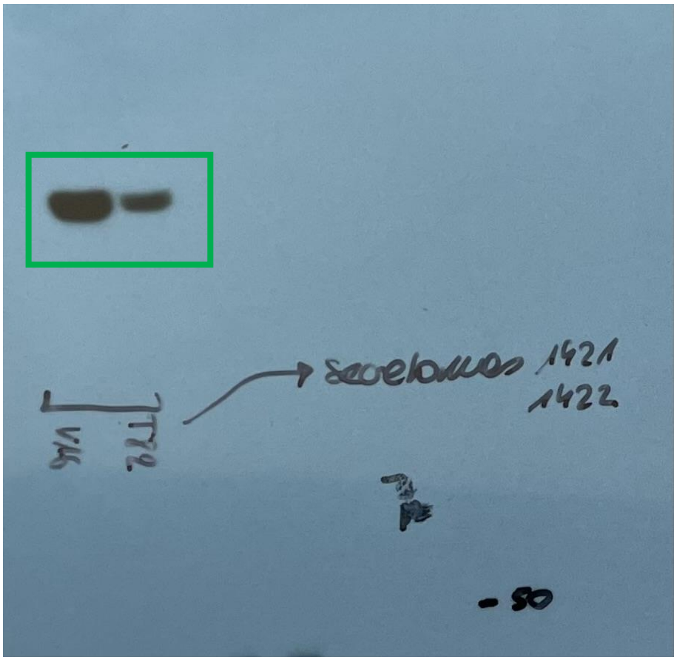

Figure 4B

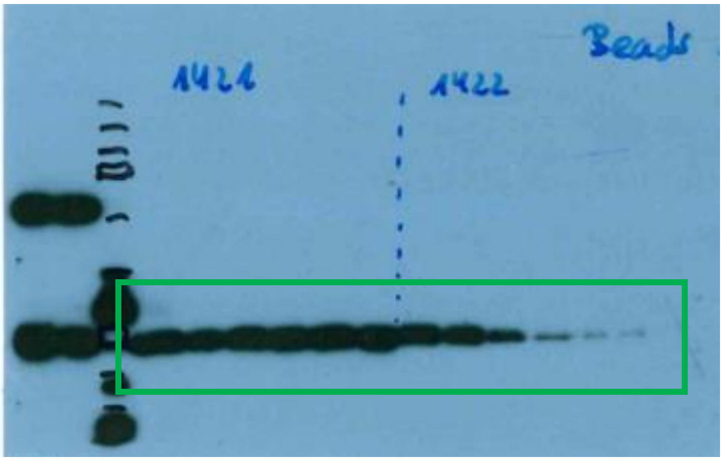

Supplement: Supplementary file 1 [file cancers-14-03858-s001.zip › cancers-1805796-supplementary.pdf]
